# Molecular Characterization of Three *GIBBERELLIN-INSENSITIVE DWARF2* Homologous Genes in Common Wheat

**DOI:** 10.1371/journal.pone.0157642

**Published:** 2016-06-21

**Authors:** XueYuan Lou, Xin Li, AiXia Li, MingYu Pu, Muhammad Shoaib, DongCheng Liu, JiaZhu Sun, AiMin Zhang, WenLong Yang

**Affiliations:** 1 The State Key Laboratory of Plant Cell and Chromosome Engineering, Institute of Genetics and Developmental Biology, Chinese Academy of Sciences, Beijing, 100101, China; 2 Graduate University of Chinese Academy of Sciences, Beijing, 100049, China; 3 The Collaborative Innovation Center for Grain crops in Henan, Henan Agricultural University, Zhengzhou, 450002, China; Murdoch University, AUSTRALIA

## Abstract

F-box protein is a core component of the ubiquitin E3 ligase SCF complex and is involved in the gibberellin (GA) signaling pathway. To elucidate the molecular mechanism of GA signaling in wheat, three homologous *GIBBERELLIN-INSENSITIVE DWARF2* genes, *TaGID2*s, were isolated from the Chinese Spring wheat variety. A subcellular localization assay in onion epidermal cells and *Arabidopsis* mesophyll protoplasts showed that *TaGID2*s are localized in the nuclei. The expression profiles using quantitative real-time polymerase chain reaction showed that *TaGID2*s were downregulated by GA_3_. The interaction between TaGID2s and TSK1 (homologous to ASK1) in yeast indicated that TaGID2s might function as a component of an E3 ubiquitin-ligase SCF complex. Yeast two-hybrid assays showed that a GA-independent interaction occurred between three TaGID2s and RHT-A1a, RHT-B1a, and RHT-D1a. Furthermore, TaGID2s interact with most RHT-1s, such as RHT-B1h, RHT-B1i, RHT-D1e, RHT-D1f, etc., but cannot interact with RHT-B1b or RHT-B1e, which have a stop codon in the DELLA motif, resulting in a lack of a GRAS domain. In addition, RHT-B1k has a frame-shift mutation in the VHIID motif leading to loss of the LHRII motif in the GRAS domain and RHT-D1h has a missense mutation in the LHRII motif. These results indicate that TaGID2s, novel positive regulators of the GA response, recognize RHT-1s in the LHRII motif resulting in poly-ubiquitination and degradation of the DELLA protein.

## Introduction

Common wheat (*Triticum aestivum* L., AABBDD) is a primary food crop worldwide. One of the most valuable wheat breeding traits is dwarfism because semi-dwarf cultivars have greater resistance potential to lodging and have stable increased yields [1]. The extensive utilization of semi-dwarf cultivars resulted in unprecedented increases in world wheat yields, driving the “green revolution” in the 1960s and 1970s [2]. The two main “green revolution” genes are *Rht-B1b* and *Rht-D1b* and encode altered forms of DELLA proteins, which function as key repressors of the gibberellin (GA) signaling pathway [3–5]. The current model of the GA signaling pathway, based on GA-GID1-DELLA, suggests that the combination of bioactive GA and its receptor GID1 promotes a conformational transition in the GRAS domain of the DELLA protein that recognizes SCF^SLY1/GID2^, which results in poly-ubiquitination and degradation of the DELLA protein via the ubiquitin-proteasome pathway, relieving DELLA-induced growth restraints and triggering GA responses [6–11].

The F-box protein of the E3 ubiquitin ligase SCF complex in the GA-GID1-DELLA module, which induces degradation of the DELLA protein, plays a pivotal role in GA signal transduction. F-box proteins contain a conserved structural F-box motif of 40–50 amino acids that functions as a protein-protein interaction site [12–13]. A large number of F-box proteins are known, such as 11 F-box proteins in budding yeast, 326 predicted in *Caenorhabditis elegans*, 22 in *Drosophila*, and at least 38 in humans [14]. Nearly 700 F-box proteins have been predicted in *Arabidopsis* and 687 potential F-box proteins have been identified in rice [15–16]. In addition to the F-box motif, F-box proteins contain a wide range of secondary motifs, including zinc fingers, cyclin domains, leucine zippers, ring fingers, tetratricopeptide repeats, and proline-rich regions [14], but the lack of a strictly conserved sequence makes it difficult to identify F-box proteins. To date, only a few F-box proteins in plants have been characterized and they were identified by studying mutants defective in specific responses.

F-box proteins are involved in plant hormone response pathways, lateral root formation, light signaling and clock control, and pollen recognition and rejection, and can be encoded by plant pathogenic microbes [17]. Since the plant hormone GA was identified as a plant growth regulator in the 1930s [18], the first recessive GA-insensitive mutation group *sly1* (*Sleepy 1*) was found by screening for suppressors of the abscisic acid (ABA)-insensitive mutant *ABI1-1* in *Arabidopsis*, as the effects of GA are often antagonistic to ABA [19]. A genetic analysis of mutants showed that SLY1 is a positive regulator of GA signaling [20–21]. The *SLY1* gene was isolated by map-based cloning using the *sly1-2* and *sly1-10* mutants [19, 22–23] and revealed that SLY1 is a putative F-box subunit of an SCF E3 ubiquitin ligase. Further studies suggested that the SCF^SLY1^ complex mediates GA-induced degradation of RGA. SLY1 interacts directly with RGA and GAI via their C-terminal GRAS domain, based on yeast two-hybrid and *in vitro* pull-down assays, further supporting the model that SCF^SLY1^ targets both RGA and GAI for degradation [24]. In addition, the SNE F-box protein replaces SLY1 during GA-induced proteolysis of RGA [25]. Coincidentally, Ashikari et al. (2003) speculated that GA-dependent degradation of SLR1 is mediated by the SCF^GID2^ complex [26]. Sasaki et al. (2003) supported this by isolating and characterizing the *gid2* rice GA-insensitive dwarf mutant [27]. Moreover, Gomi et al. (2004) clarified that phosphorylated SLR1 is bound by the SCF^GID2^ complex through an interaction between GID2 and SLR1, triggering ubiquitin-mediated degradation of SLR1 [28]. Although studies in *Arabidopsis* and rice have revealed how the GA signal is perceived and transmitted downstream, the molecular mechanism of GA signaling in wheat is unknown. In this study, we isolated three homologous *GID2* genes and analyzed their biological and molecular properties. The results suggest that TaGID2, which is a component of the SCF complex, interacts with RHT-1 via the LHRII motif of a DELLA protein.

## Materials and Methods

### Plant growth and treatment

Plant material was grown in a greenhouse at 23°C under a 16 h light/8 h dark regime for 2 weeks. Young leaves from the Chinese Spring wheat cultivar, 21 nulli-tetrasomic lines, and 11 deletion lines of Chinese Spring were collected to isolate genomic DNA using the cetyltrimethylammonium ammonium bromide method [29–30]. Young leaves from Chinese Spring and the wild wheat diploid relatives *Triticum urartu* (AA), *Aegilops speltoides* (BB), and *Aegilops tauschii* (DD) were sampled for RNA extraction using the RNeasy Plant Mini Kit (QIAGEN, Hilden, Germany), and first-strand cDNA was synthesized using the FastQuant RT Kit (with gDNase) (TIANGEN, Beijing, China) according to the manufacturer’s instructions. Various wheat tissues, including young spikes, flag leaves, peduncles, the third and fourth internodes, and roots from Chinese Spring at the heading stage and seedlings of Chinese Spring treated with 30 μmol·L^-1^GA_3_ or ddH_2_O (control) for 2 weeks were collected for RNA isolation.

### Cloning the wheat *GID2* and *TSK1* genes

To isolate the homologous *GID2* genes in wheat, we selected a series of wheat expressed sequence tags (ESTs), which were highly similar to *OsGID2* (AB100246), from the TIGR wheat EST database (http://blast.jcvi.org/euk-blast/index.cgi?-project=tae1) and designed a pair of Contig1.GID2.F/R primers (Table 1) by assembling the sequences. This primer pair was used to clone the *GID2* genes in the genomic DNA and cDNA of Chinese Spring and the wild wheat diploid relatives. Amplifications were performed in a 20 μL reaction volume containing 100 ng DNA template, 0.4 μM forward and reverse primers, 1 U TaKaRa LA Taq (Takara, Biotechnology Co., Ltd, Dalian, China), 1× GC buffer I, and 0.4 mM of each dNTP under the following conditions: 94°C for 5 min, followed by 35 cycles of 94°C for 30 sec, 58°C for 30 sec, 72°C for 1 min, and a final extension at 72°C for 10 min. The polymerase china reaction (PCR) products were cloned into the pGEM-T easy vector (Promega, Madison, WI, USA), introduced into *Escherichia coli*, and 10 positive independent clones were sequenced commercially. DNAMAN and Clustal W were used for the sequence alignment and Clustal W and MEGA ver. 5.1 were used to draw the phylogenetic trees. In addition, to demonstrate whether *TaGID2*s play roles in the SCF ubiquitin-ligase complex, we designed the TSK1.F/R primers (Table 1) according to the complete coding sequence (CDS) of the *T*. *aestivum* SKP1/ASK1-like protein (AY316293), which was cloned by Li et al. (2006) [31]. PCR was carried out using Chinese Spring cDNA as the template, and we obtained a single clone called *TSK1*.

**Table 1:** Polymerase chain reaction primers used in this study.

Primer	Forward primer sequence	Reverse primer sequence
Contig1.GID2.F/R	5′-TGTCCATCTCTCCTCGTCAGATCCATC-3′	5′-CGGTGCGCTGGACTGGTTGA-3′
TSK1.F/R	5′-CGCGACTAGAGTTTCCTCGCTAGGG-3′	5′-ACGATTAAGATTCAGTTTGACAAGT-3′
TaGID2-1LF/1LR	5′-ACGACGAGCACAGGTGAGCAC-3′	5′-CAACAACCAGTATGGATCATGAA-3′
TaGID2-2LF/2LR	5′-AGCACGAGCACAGGTGAGTAT-3′	5′-CCTTGTTCTTTGGCCAGCTT-3′
TaGID2-3LF/3LR	5′-AGCACGAGCACAGGTGAGTAT-3′	5′-TGGCCAAAAGAACAGAGCATA-3′
TaGID2-A-*Hin*dIII.F/*Bam*HI.R	5′-AAGCTT ATGAAGTGCCCTTCCGATTCCTC-3′	5′-GGATCC CTGGGACGGCGAGGGAG-3′
TaGID2-B-*Hin*dIII.F/*Bam*HI.R	5′-AAGCTT ATGAAGCGCCCTTCCGGTT-3′	5′-GGATCC CTGGGATGGCGAGGGAG-3′
TaGID2-D-*Hin*dIII.F/*Bam*HI.R	5′-AAGCTT ATGAAGTGCCCTTCCGATTCCTC-3′	5′-GGATCC CTGGGACGGCGCGGGAG-3′
TaGID2-A-*attB*.F/R	5′-GGGGACAAGTTTGTACAAAAAAGCAGGCTGC ATGAAGTGCCCTTCCGATTCCTC-3′	5′-GGGGACCACTTTGTACAAGAAAGCTGGGTC CTGGGACGGCGAGGGAG-3′
TaGID2-B-*attB*.F/R	5′-GGGGACAAGTTTGTACAAAAAAGCAGGCTGC ATGAAGCGCCCTTCCGGTT-3′	5′-GGGGACCACTTTGTACAAGAAAGCTGGGTC CTGGGATGGCGAGGGAG-3′
TaGID2-D-*attB*.F/R	5′-GGGGACAAGTTTGTACAAAAAAGCAGGCTGC ATGAAGTGCCCTTCCGATTCCTC-3′	5′-GGGGACCACTTTGTACAAGAAAGCTGGGTC CTGGGACGGCGCGGGAG-3′
TaGID2-A-EF/ER	5′-GCATCCTCGTCCTCACAG-3′	5′-GAAAGAGAGCACGACGTCG-3′
TaGID2-B-EF/ER	5′-TCACAGCCTCCACCGGC-3′	5′-GAACGGGAGCACGACGG-3′
TaGID2-D-EF/ER	5′-ACAGCCTCCGCCGCAGCA-3′	5′-CCTCCTCCTCCCCCGTTC-3′
Ta4045-EF/ER	5'-CCTGCCCCGTACAACCTTGAG-3'	5'-CACCGTTGCGATAGTCCTGAAAC-3'
TSK1-*Nde*I.F/*Eco*RI.R	5′-CATATG ATGGCGGCCGCGGGAGAC-3′	5′-GAATTC CTACTCAAAGGCCCACTGGTTCTC-3′
TaGID2-A1-*Nde*I.F/*Eco*RI.R	5′-CATATG ATGAAGTGCCCTTCCGATTCCTC-3′	5′-GAATTC TCACTGGGACGGCGAGGGAG-3′
TaGID2-B1-*Nde*I.F/*Eco*RI.R	5′-CATATG ATGAAGCGCCCTTCCGGTT-3′	5′-GAATTC TCACTGGGATGGCGAGGGAG-3′
TaGID2-D1-*Nde*I.F/*Eco*RI.R	5′-CATATG ATGAAGTGCCCTTCCGATTCCTC-3′	5′-GAATTC TCACTGGGACGGCGCGGGAG-3′
Rht-A1-*Nde*I.F/*Eco*RI.R	5′-CATATG AAGCGCGAGTACCAGGAC-3′	5′-GAATTC TCAAAACTCGCGATCACG-3′
Rht-B1-*Nde*I.F/*Eco*RI.R	5′-CATATG AAGCGCGAGTACCAGGACG-3′	5′-GAATTC TCAAAACTCGCGGTCACGG-3′
Rht-D1-*Nde*I.F/*Eco*RI.R	5′-CATATG AAGCGGGAGTACCAGGACG-3′	5′-GAATTC TCAAAACTCGCGAGATCACG-3′
TaGID1A-*Eco*RI.F/*Bam*HI.R	5′- GAATTC ATGGCCGGCAGCGACGAG -3′	5′- GGATCC CTACAGGAGGTTAGCTCGGACGA -3′
TaGID2A-*Not*I.F/*Bgl*II.R	5′- GCGGCCGC ATGAAGTGCCCTTCCGATTCCTC -3′	5′- AGATCT TCACTGGGACGGCGAGGGAG -3′

### Physical localization assay

Three sets of gene-specific primers, TaGID2-1LF/1LR, TaGID2-2LF/2LR, and TaGID2-3LF/3LR (Table 1), were used to amplify the genomic DNA of Chinese Spring, 21 nulli-tetrasomic lines, and 11 deletion lines of Chinese Spring to confirm the location of *TaGID2*s on the wheat chromosomes. The PCR products were resolved by 1.5% agarose gel electrophoresis.

### Real-time PCR analysis

Quantitative real-time PCR (qRT-PCR) was performed to measure transcript levels of *TaGID2*s using the LightCycler 480 system (Roche, Indianapolis, IN, USA). SYBR Green I Master (Roche) was used according to the manufacturer’s protocol. Three pairs of gene-specific primers, TaGID2-A-EF/ER, TaGID2-B-EF/ER, and TaGID2-D-EF/ER (Table 1), were used for the gene expression analysis, and the *Ta4045* gene was used as the reference [32]. The expression profiles of the three *TaGID2*s in various wheat organs at the heading stage and their response to exogenous GA were investigated according to the description of Li et al. (2013a) [33]. All qRT-PCR experiments were performed independently using three biological and three technical replicates.

### Subcellular localization assay

The subcellular localization assay was performed by transiently expressing the TaGID2-green fluorescent protein (GFP)/yellow fluorescent protein (YFP) fusion protein in onion epidermal cells and *Arabidopsis* protoplasts, respectively. The GFP fusion proteins of TaGID2A-GFP, TaGID2B-GFP, and TaGID2D-GFP were constructed using PCR combined with the restriction enzyme digestion method and the TaGID2-A-*Hin*dIII.F/*Bam*HI.R, TaGID2-B-*Hin*dIII.F/*Bam*HI.R, and TaGID2-D-*Hin*dIII.F/*Bam*HI.R primers (Table 1). The TaGID2-GFP fusion protein was transformed into onion epidermal cells by particle bombardment with a PDS-1000/He system (Bio-Rad, Hercules, CA, USA) according to the method of Oh et al. (2008) using the pJIT-163-hGFP plasmid as a positive control [34]. The complete CDS regions of the *TaGID2*s were amplified with the TaGID2-A-*attB*.F/R, TaGID2-B-*attB*.F/R, and TaGID2-D-*attB*.F/R primers (Table 1) and cloned into the pEarleyGate101 vector using the Gateway-compatible vector cloning system to generate the TaGID2A-YFP, TaGID2B-YFP, and TaGID2D-YFP constructs [35]. *Arabidopsis* mesophyll protoplast cells were isolated and transformed using the PEG transformation method as described by Yoo et al. (2007) [36]. The pA7-YFP plasmid was introduced into the protoplasts as a positive control. After 18–24 h incubation, the GFP/YFP fluorescence emissions from living cells were observed under a confocal microscope (Zeiss LSM 710 NLO; Carl Zeiss, Oberkochen, Germany) at excitation wavelengths of 488/514 nm and emission wavelengths of 506–538/527 nm.

### Yeast two-hybrid assay

The yeast two-hybrid assay was performed to detect the interactions between TaGID2 and TSK1/ RHT-1s, as well as the interactions between TaGID1s and RHT-1s *in vitro*. The CDSs of *TaGID1*s, *TSK1*, *TaGID2*s, *SLN1*, and *Rht-1*s were ligated into the pGADT7 and pGBKT7 vectors (Clontech, Mountain View, CA, USA) [37–38], using the *Nde*I and *Eco*RI sites to generate the pGADT7-*TSK1*, pGADT7-*SLN1*, and pGADT7-*RHT1*s prey plasmids and the pGBKT7-*TaGID1*s, pGBKT7-*TaGID2*s bait plasmids, respectively. The gene-specific primers are shown in Table 1. The AD and BK fusion constructs were co-transformed into *Saccharomyces cerevisiae* strain AH109, and the transformants were screened on SD/-Leu-Trp media. The positive transformants were further streaked on SD/-Leu-Trp-His-Ade media with or without 100 μmol·L^-1^ GA_3_. The plate assay was carried out according to the Yeast Protocol Handbook (Clontech).

### Bimolecular fluorescence complementation (BiFC) assay

The BiFC assay was performed to detect the interactions between TaGID1-A1 and RHT-1s *in vivo*. The complete CDS regions of *TaGID1-A1* and *Rht-1*s were amplified with the TaGID1-A-*attB*.F/R, Rht-B1-*attB*.F/R, and Rht-D1-*attB*.F/R primers (Table 1) and cloned into the pX-nYFP and pX-cCFP vectors using the Gateway technology to generate the TaGID1A-nYFP, TaGID1A-cCFP, RHT1s-nYFP, and RHT1s-cCFP constructs. *Arabidopsis* mesophyll protoplasts were isolated and co-transformed using the PEG transformation method as described by Yoo et al. (2007) [36]. The pA7-YFP plasmid was introduced into the protoplasts as a positive control. After 18–24 h incubation, YFP fluorescence emission in living cells was observed under a confocal microscope (Zeiss LSM 710 NLO) at an excitation wavelength of 514 nm and emission wavelength of 527 nm. Chloroplast autofluorescence was observed at an excitation wavelength of 488 nm and emission wavelengths of 664–696 nm.

### Western blot analysis and co-immunoprecipitation

The fourth internodes were sampled at the heading stage, batch-frozen in liquid N_2_, and ground into powder using a mortar and pestle to extract total soluble protein. The powder (1 g) was resuspended in 1 mL extraction buffer (50 mM Tris-HCl pH 7.5, 150 mM NaCl, 1 mM DTT, 1 mM EDTA pH 8.0, 10% glycerol, 1% Triton X-100, and protease inhibitor cocktail; Roche), the suspension was vortexed continuously at 100 rpm for 30 min, and then centrifuged at 16,000 g for 30 min. The supernatant was collected and resolved by 10% acrylamide gel electrophoresis, and electro-transferred to PVDF membranes. The membranes were blocked for 2–3 h at room temperature in TBST (20 mM Tris-HCl, pH 8.0, 137 mM NaCl, 0.05% [v/v] Tween 20) containing 5% (w/v) nonfat dry milk. The membranes were incubated with TaGID2 polyclonal antibody (1:1,000; CWBIO Co. Ltd, Beijing, China) for 12 h at 4°C. All dilutions were done in TBST containing 5% (w/v) nonfat dry milk. The blots were washed five times in TBST for 6 min each before incubation with the secondary horseradish peroxidase-conjugated goat α-rabbit antibody (1:10,000; cat. no. CW0103 CWBIO). The blots were washed five times with TBST for 6 min each, the bound antibody was detected with the eECL Western Blot Kit (cat. no. CW0049A; CWBIO), and exposed to X-ray film (Kodak-Biomax Light; Kodak Inc., Rochester, NY, USA).

Protein extracts were prepared as described above for the Western blot analysis. The resulting supernatants were incubated with DELLA monoclonal antibodies (1:100; Abmart Co. Ltd., Shanghai, China) for 12 h at 4°C. Then, 60 μl Protein G Agarose beads (Millipore, Billerica, MA, USA) were added to each tube and vortexed continuously at 70 rpm for 2 h. The antibody-antigen complexes were precipitated by centrifugation for 5 min at 2,000 g, washed four times with 1 mL PBS, and eluted from the beads by boiling in sodium dodecyl sulfate-polyacrylamide gel electrophoresis sample buffer for 5 min. Equal quantities of immunoprecipitated and unbound proteins were resolved on 10% acrylamide gels. Protein immunoblots were carried out as described for the Western blot analysis.

### Yeast three-hybrid assay

The yeast three-hybrid assay was performed using pGADT7 and pBridge (Clontech) as expression vectors to detect the interaction between TaGID1-DELLA-TaGID2 *in vitro*. The pGADT7-*SLN1* and pGADT7-*RHT1*s plasmids were used as prey. The pBr-TaGID1A-TaGID2A bait plasmids were generated by using the *Bam*HI and *Eco*RI sites in MCSI for TaGID1s and the *Not*I and *Bgl*II site in MCSII for TaGID2 using the specific primers shown in Table 1. The AD and pBr fusion constructs were co-transformed into yeast strain HF7c, the transformants were screened on SD/-Met-Leu-Trp media, and the positive transformants were further streaked on SD/-Met-Leu-Trp-His media containing 20 mM 3-amino-1, 2, 4-triazole with or without 100 μmol·L^-1^ GA_3_.

## Results

### *TaGID2*s are orthologous to *OsGID2*

**Fig 1:**
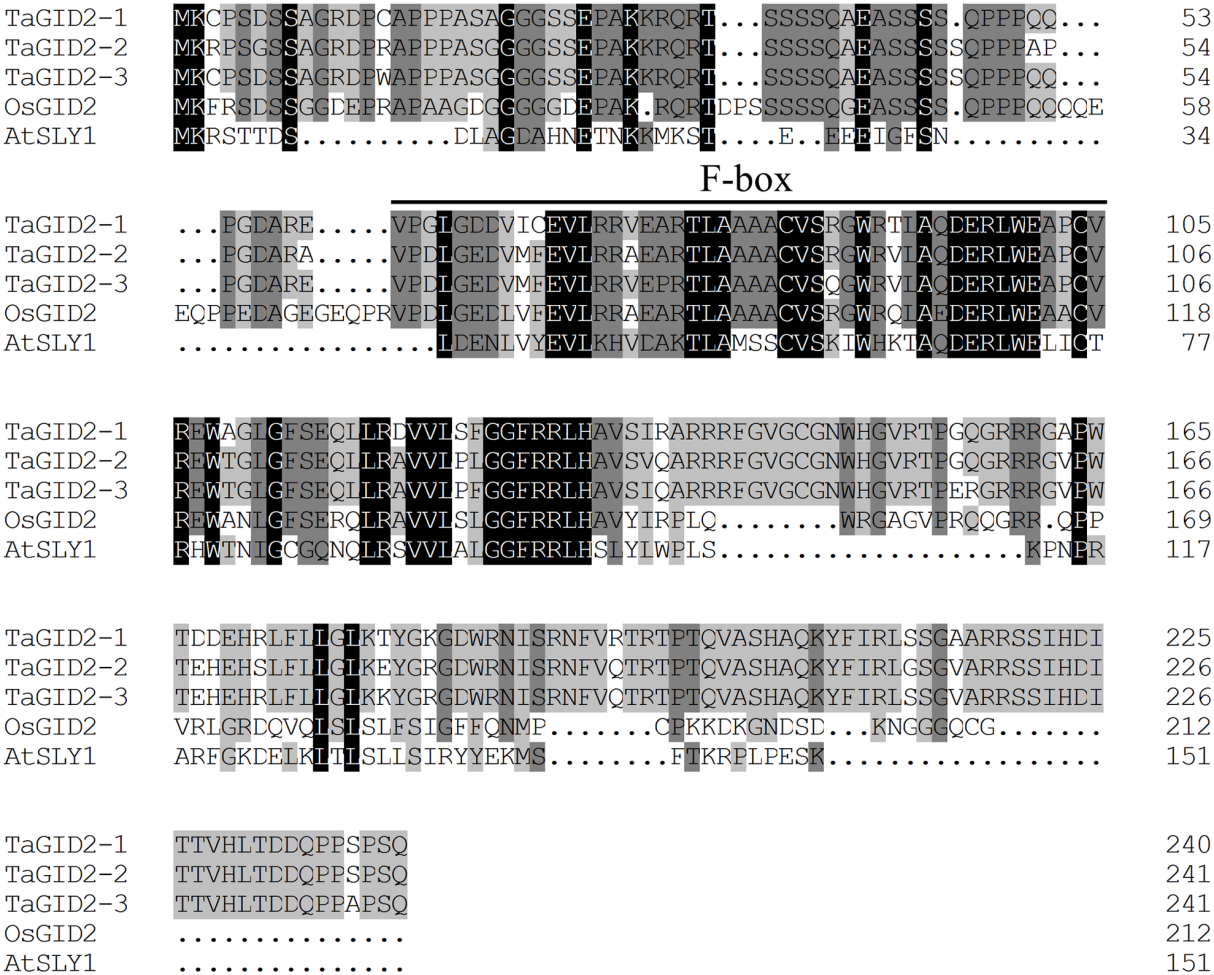
Multiple sequence alignment of the GID2/SLY1 proteins from wheat, rice, and *Arabidopsis*. The conserved F-box domain is indicated by black line.

To elucidate the function of F-box proteins in GA signal transduction, three homologous genes were cloned from the cDNA of the Chinese Spring wheat variety using PCR and the Contig1.GID2.F/R primers. The cDNA sequences of the three genes were 812, 815, and 815 bp long, respectively, and contained 723, 726, and 726 bp CDSs, respectively, and partial 5′- and 3′-untranslated regions (Table 2). They encoded 240, 242, and 241 amino acid residues with molecular weights of 26.24, 26.26, and 26.69 KDa, with pIs of 10.36, 11.12, and 10.53, respectively. A BLAST search revealed that the predicted protein sequences were homologous with the F-box protein family [15]. Comparison of the primary structures of the three predicted proteins with OsGID2 and AtSLY1 revealed that three putative amino acid sequences shared 45.70%, 47.27%, and 45.53% sequence identity with OsGID2 and only 19.58%, 21.16%, and 21.58% sequence identity with AtSLY1, respectively, whereas their F-box domains had similarities of 80.85%, 89.36%, and 82.98% with OsGID2 and 48.94%, 48.94%, and 48.94% with AtSLY1, respectively, indicating that the three genes in wheat are orthologous to *OsGID2* and *AtSLY1*. Thus, the three genes were named *TaGID2-1*, *TaGID2-2*, and *TaGID2-3* (Genbank accession numbers KU857036–KU857038; Fig 1).

**Table 2:** Analysis of the composition and physicochemical characteristics of the cDNA sequences and deduced amino acid sequences of the *TaGID2* genes.

Characters	*TaGID2-1*	*TaGID2-2*	*TaGID2-3*
cDNA sequence length (bp)	812	815	815
CDS length (bp)	723	726	726
Amino acid residue number	240	241	241
Molecular weight (kDa)	26.24	26.26	26.69
pI	10.36	11.12	10.53
Acidic amino acid residue number	23	21	23
Basic amino acid residue number	36	35	35
Instability index	67.00	70.00	70.79
Aliphatic index	62.25	63.98	62.74
Grand average of hydropathicity	−0.672	−0.644	−0.692

Analysis of the conserved domain (http://www.ncbi.nlm.nih.gov/Structure/cdd/wrpsb.cgi) showed that all three TaGID2s have F-box and SANT conserved domains. The sequence alignment of OsGID2 and TaGID2s (Fig 1) indicated that TaGID2-1, TaGID2-2, and TaGID2-3 share five motifs, including the VR1, F-box, GGF, VR2, and LSL motifs as in OsGID2. The physicochemical property analysis using the ProtParam tool (http://web.expasy.org/protparam/) showed that the three TaGID2s were unstable hydrophilic and alkaline lipid soluble proteins. The SignalP 4.1 Server (http://www.cbs.dtu.dk/services/SignalP/) and TMHMM Server v. 2.0 (http://www.cbs.dtu.dk/services/TMHMM/) results predicted that the TaGID2s were non-secretory, non-transmembrane cytoplasmic proteins. The phylogenetic tree of the GID2 proteins (S1 Fig) was divided into two major groups of dicotyledons and monocotyledons. The GID2 proteins from dicotyledons, such as *Arabidopsis*, soybean, grape, and sunflower, formed one cluster, and the GID2 proteins of monocotyledons, such as, rice, wheat, and maize, formed another cluster. The SmGID2a and SmGID2c were clustered separately. TaGID2 had a close evolutionary relationship with AetGID2 and OsGID2, whereas the relationship was more distant with AtSLY1 and BnSLY1, and even more distant with SmGID2, indicating that the evolutionary relationship among the GID2 proteins is consistent with their taxonomic distance.

The corresponding genomic DNA sequences of the three *TaGID2*s were isolated from the Chinese Spring wheat variety (Genbank accession numbers KU857045–KU857047; S2 Fig) Their lengths were 1,085, 1,160, and 1,160 bp, and they shared high similarity with each other. All of the DNA sequences had two exons and one intron. The intron in *TaGID2-1* occurred between nucleotides 509 and 510 in the CDS sequence, whereas both introns of *TaGID2-2* and *TaGID2-3* were situated at nucleotides 512 and 513 of the CDS sequence, and their lengths were 273, 345, and 345 bp, respectively.

### Three *TaGID2*s were located on the short arms of group 3 chromosomes

**Fig 2:**
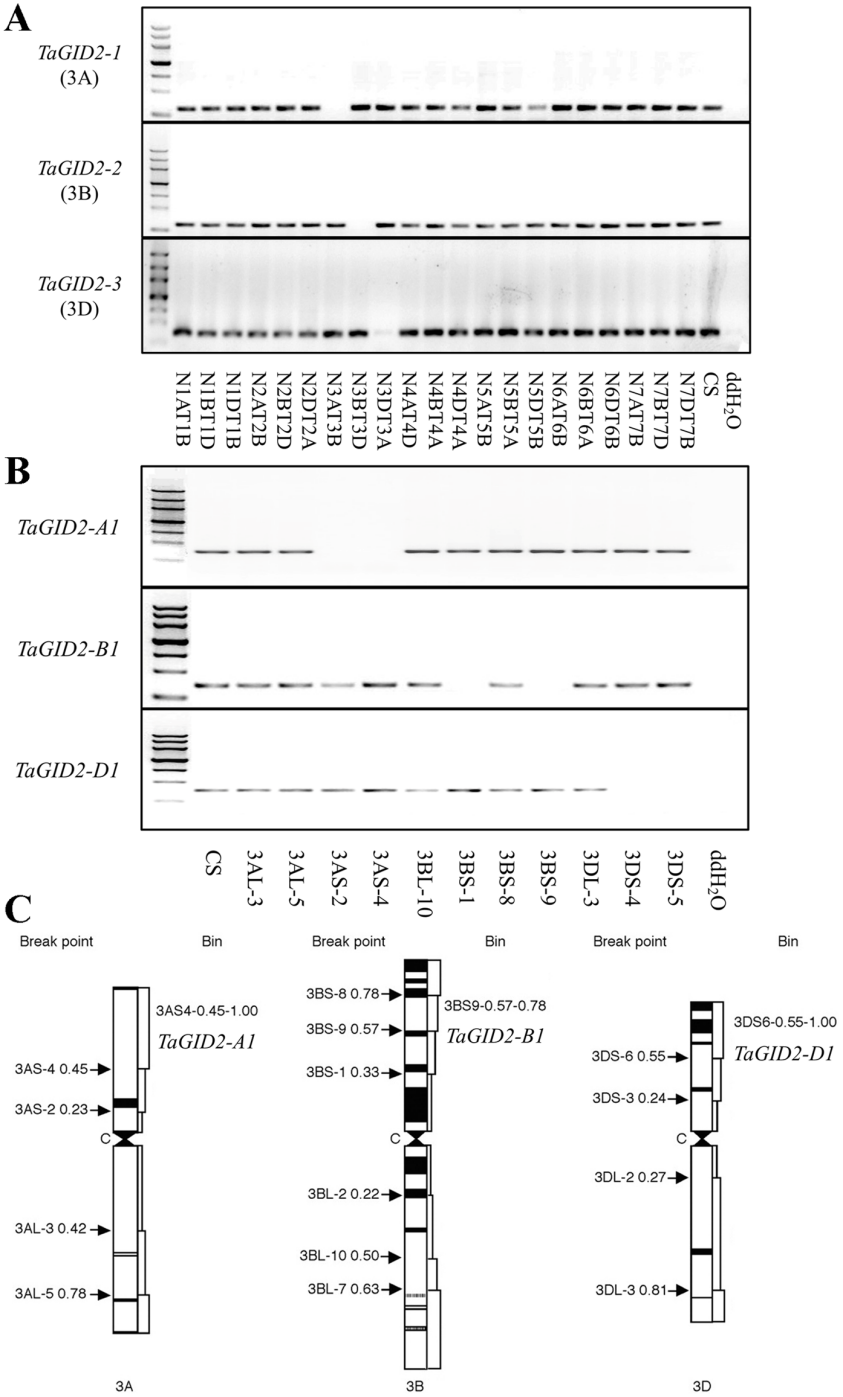
Physical localization assay of the three *TaGID2s*. (A) Chromosome location of the three *TaGID2*s, the genomic DNA of a set of Chinese Spring nulli-tetrasomic lines was amplified using the homolog-specific primers of *TaGID2-1*, *TaGID2-2*, and *TaGID2-3*, with ddH_2_O as a control. (B) Chromosomal bin location of the three *TaGID2*s, products amplified from 11 deletion lines of Chinese Spring using the homolog-specific primers of *TaGID2-1*, *TaGID2-2*, and *TaGID2-3* were detected by 1.5% agarose gel, with ddH2O as a control. (C) Position of the three *TaGID2*s in chromosomes 3A, 3B and 3D, the names and breakpoints of the deletion lines are shown on the left, and the chromosome bins and *TaGID2* genes are shown on the right.

We amplified 21 nulli-tetrasomic lines of Chinese Spring (Fig 2A) and found that the *TaGID2-1* gene specific primers amplified nothing in N3AT3B, the *TaGID2-2* gene specific primers did not produce a product in N3BT3D, and the *TaGID2-3* gene specific primers failed to produce a product in N3DT3A, indicating that the three *TaGID2*s were individually assigned to the 3A, 3B, and 3D wheat chromosomes. Thus, they were renamed *TaGID2-A1*, *TaGID2-B1*, and *TaGID2-D1*, respectively. In addition, we determined the location of the *TaGID2* chromosome bin (Fig 2B) using 11 deletion lines from group 3 chromosomes [39]. Among the four 3A deletion lines, the *TaGID2-A1* gene specific primers did not yield a product in 3AS-2 and 3AS-4, the *TaGID2-B1* gene specific primers did not yield a product in 3BS-1 and 3BS-9 in four 3B deletion lines, and the *TaGID2-D1* gene specific primers failed to amplify 3DS-4 and 3DS-5 in the three 3D deletion lines, demonstrating that the three *TaGID2*s are located in chromosome bins 3AS4-0.45–1.00, 3BS9-0.57–0.78, and 3DS4-0.59–1.00, respectively (Fig 2C).

The orthologous *GID2* genes were cloned from wild wheat diploid relatives using cDNA as templates to investigate the evolution of the three *TaGID2*s (S3 Fig). Two genes were obtained from *Triticum urartu* (AA) and *Aegilops tauschii* (DD) and named *TuGID2* and *AetGID2*, respectively (Genbank accession numbers KU857039 and KU857043, respectively). Three genes were obtained from *Aegilops speltoides* (BB) and named *AesGID2-1*, *AesGID2-2*, and *AesGID2-3*, respectively (Genbank accession numbers KU857040-KU857042). The respective CDS lengths of *TuGID2*, *AesGID2-1*, *AesGID2-2*, *AesGID2-3*, and *AetGID2* were 723, 723, 726, 726, and 726 bp. These GID2s had high sequence identities with the TaGID2s. A phylogenetic analysis of the putative GID2 proteins from common wheat and its relatives (S3 Fig) showed that TaGID2-A1 was clustered with TuGID2, TaGID2-B1 was clustered with AesGID2-1, AesGID2-2, and AesGID2-3, and TaGID2-D1 was clustered with AetGID2, which are consistent with the chromosome locations of the three *TaGID2*s.

### GA downregulates *TaGID2*s expression levels in wheat

**Fig 3:**
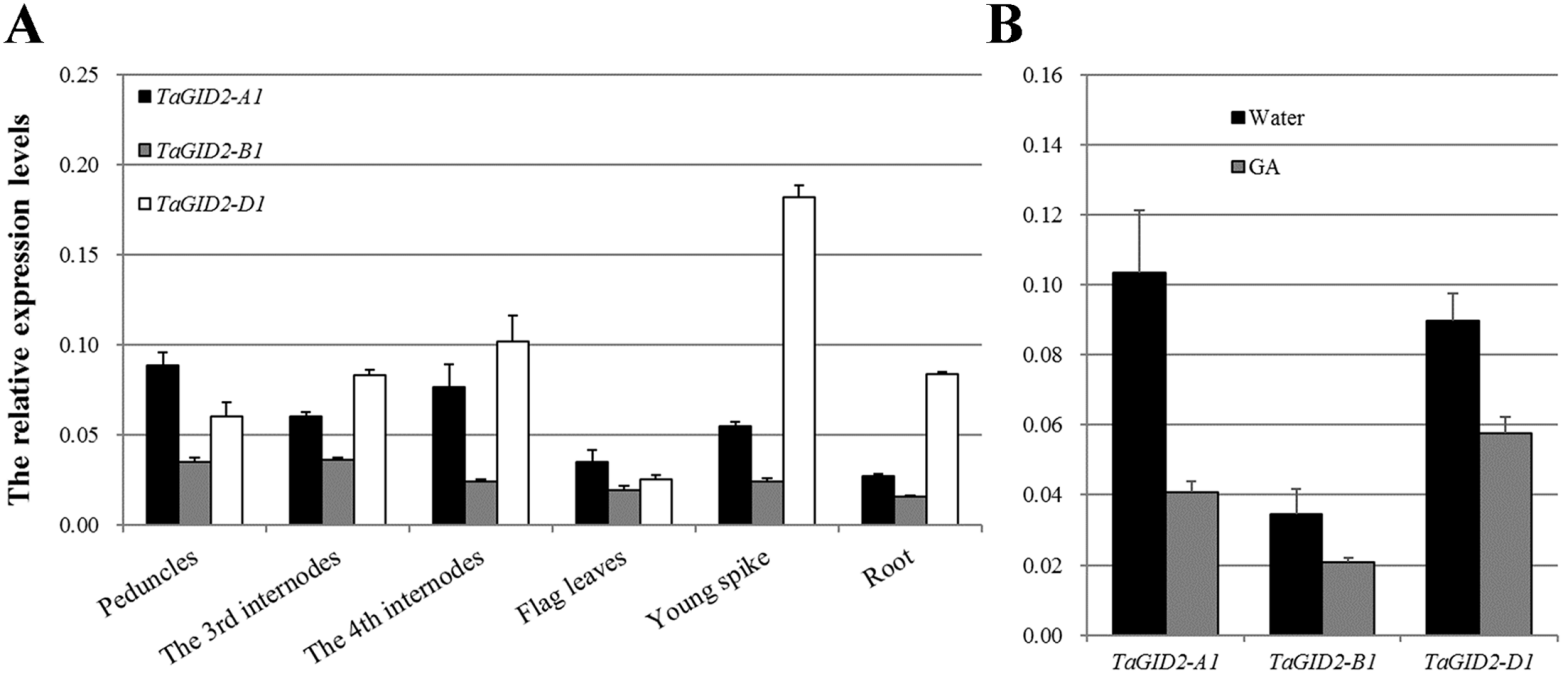
*TaGID2* gene expression analysis in wheat. (A) Expression profiles of the *TaGID2* genes in wheat tissues including peduncles, the third and fourth internodes, flag leaves, young spikes and roots. (B) Feedback regulation of the *TaGID2*s in seedlings after 30 μM GA_3_ treatment.

Similar expression patterns were observed for the three *TaGID2*s in various wheat organs (Fig 3A) at the heading stage. *TaGID2*s were constitutively transcribed in young spikes, flag leaves, peduncles, the third and fourth internodes, and roots from Chinese Spring. However, their expression levels in six organs were different, and they were preferentially expressed in young spikes and elongating stems, such as the third and fourth internodes, but transcribed less in flag leaves and roots. Among the three *TaGID2*s, *TaGID2-B1* was expressed at the lowest level, and *TaGID2-D1* was transcribed the most in the six organs, except peduncles and flag leaves. Furthermore, *TaGID2*s were downregulated in seedlings treated with 30 μmol·L^-1^ GA_3_ (Fig 3B), suggesting feedback regulation of *TaGID2*s by GA in wheat.

### TaGID2s form an SCF complex by interacting with TSK1

**Fig 4:**
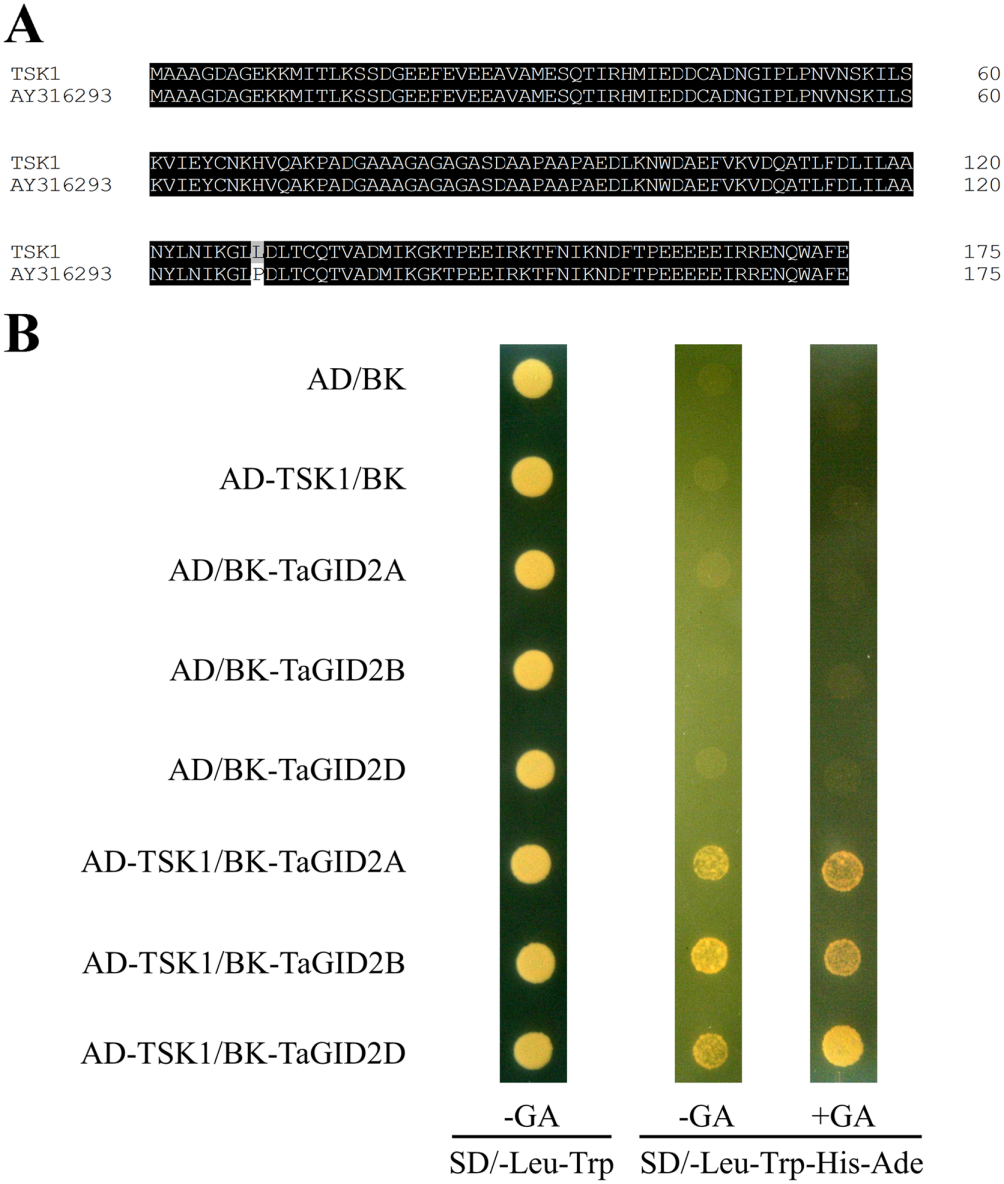
Interactions among TaGID2 and TSK1 proteins in yeast cells. (A) Amino acid sequence alignment of TSK1 from Chinese Spring with AY316293 from Jingdong 1. (B) Yeast two hybrid assay between TaGID2 and TSK1, TSK1 was used as prey, and TaGID2-A1, TaGID2-B1, and TaGID2-D1 were used as baits, the AD and BK constructs were co-transformed into the yeast strain AH109, the transformants were grown on the SD/-Leu-Trp media (control) and the SD/-Leu-Trp-His-Ade media with or without 100 μM GA_3_, all experiments were conducted three times, and five clones were used each time.

The *Arabidopsis ASK1* homologous gene *TSK1* (Genbank accession number KU857044) was cloned from the Chinese Spring wheat variety to clarify the role of TaGID2 in formation of the SCF complex, which has only two single nucleotide polymorphisms (SNPs) with AY316293 in the CDS (Fig 4A) [31]. *TSK1* and AY316293 could be the same gene or a different copy of the same gene. The yeast two-hybrid assay showed that TSK1 interacted with TaGID2s directly in a GA-independent way (Fig 4B), indicating that TaGID2s might function as a component of an SCF E3 ubiquitin-ligase complex, triggering poly-ubiquitination and degradation of DELLA proteins.

### TaGID2s interact with RHT-1 in the nuclei

**Fig 5:**
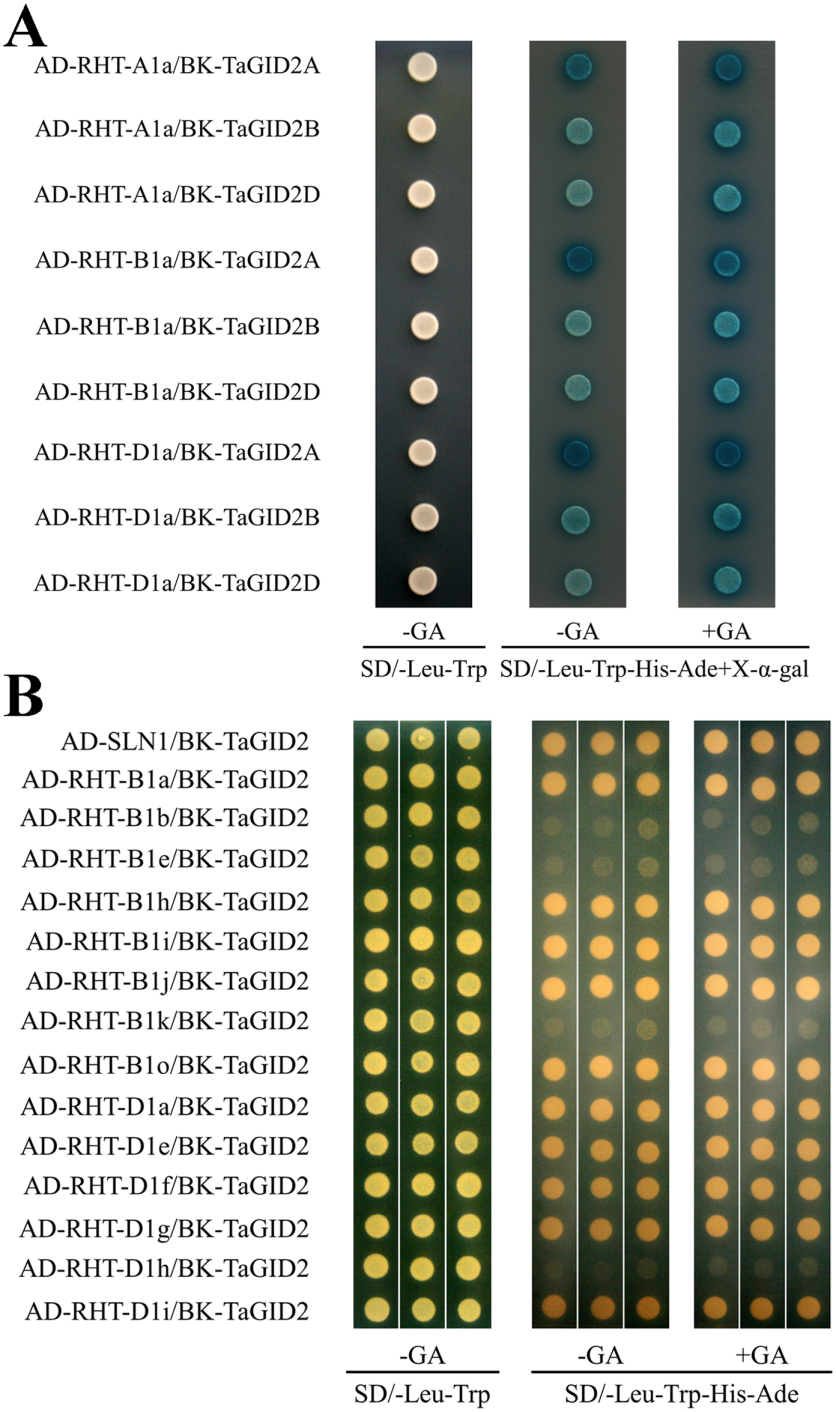
Interaction between TaGID2 and RHT-1 in yeast cells. (A) TaGID2s interact with RHT-1 in yeast cells, TaGID2-A1, TaGID2-B1, and TaGID2-D1 were used as baits, and RHT-A1a, RHT-B1a, and RHT-D1a were used as preys, the AD and BK constructs were co-transformed into the yeast strain AH109, the transformants were grown on the SD/-Leu-Trp media (control) and the SD/-Leu-Trp-His-Ade media containing 20 μg/mL X-α-Gal with or without 100 μM GA_3_, all experiments were conducted three times, and five clones were used each time. (B) TaGID2s interact with allelic variations of RHT-1 in the LHRII motif, SLN1 and RHT-1s were used as preys, TaGID2-A1, TaGID2-B1, and TaGID2-D1 were used as baits from left to right in each block, and the AD and BK constructs were co-transformed into the yeast strain AH109, the transformants were grown on the SD/-Leu-Trp media (control) and the SD/-Leu-Trp-His-Ade media with or without 100 μM GA_3_, all experiments were conducted three times, and five clones were used each time.

The yeast two-hybrid assay showed that GA-independent interactions occurred among the three TaGID2s and RHT-A1a, RHT-B1a, and RHT-D1a, demonstrating that RHT-1s are targets of TaGID2s. Moreover, among the nine TaGID2/RHT-1 combinations, TaGID2-A1 exhibited relatively stronger activities than those of TaGID2-B1 and TaGID2-D1 when interacting with the same RHT-1, suggesting it has stronger affinity to DELLA proteins (Fig 5A). To further verify the TaGID2 and RHT-1 interacting region, the CDS of SLN1 and 14 allelic variations of *Rht-1* were constructed into pGADT7 and the three *TaGID2*s into pGBKT7 for yeast two-hybrid assays. The results showed that TaGID2s interacted with RHT-B1a, RHT-B1h, RHT-B1i, RHT-B1j, RHT-B1o, RHT-D1a, RHT-D1e, RHT-D1f, RHT-D1g, and RHT-D1i in a GA-independent manner, but did not interact with RHT-B1b, RHT-B1e, RHT-B1k, or RHT-D1h (Fig 5B). The reason may be a stop codon in the RHT-B1b and RHT-B1e DELLA motif resulting in a lack of the GRAS domain, which would prevent interaction with TaGID2s. Although RHT-B1k and RHT-D1g frameshifts occurred in the GRAS domain, the RHT-B1k frameshift occurred in the VHIID motif, causing loss of the LHRII motif, which could prevent RHT-B1k from interacting with TaGID2s, whereas the RHT-D1g frameshift occurred in the PFYRE motif, giving rise to the interaction between RHT-D1g and TaGID2s. The RHT-D1e and RHT-D1h missense mutations both occurred in the LHRII motif, but only the RHT-D1h mutation resulted in loss of the interaction with DELLA-GID2, which was consistent with the evaluation by the SIFT program that RHT-D1h was predicted to have a damaging effect on the protein. As a consequence, the interaction between TaGID2 and DELLA occurred in the LHRII motif.

**Fig 6:**
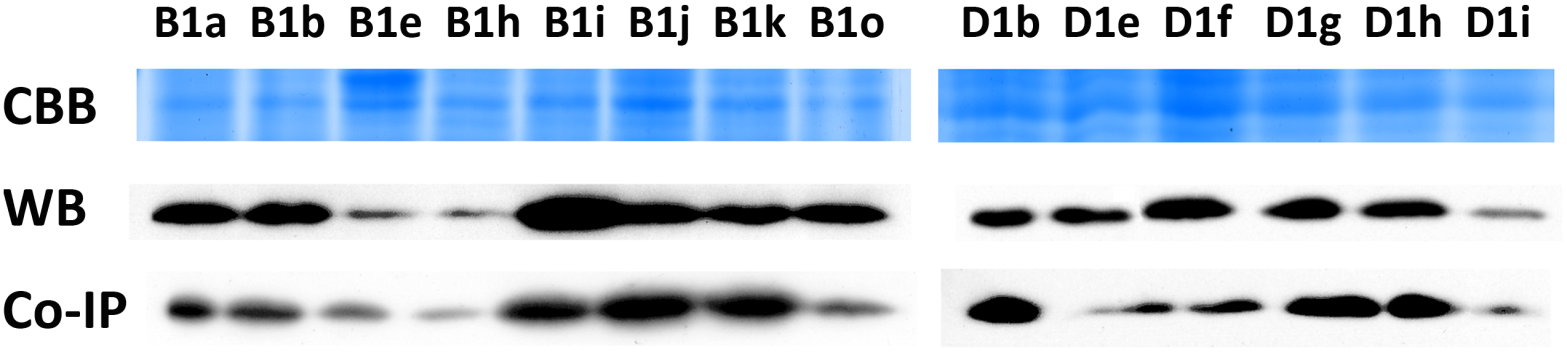
Interactions between TaGID2 and RHT-1 in wheat. CBB: Sodium dodecyl sulfate-polyacrylamide gel electrophoresis gel stained with Coomassie Brilliant Blue; WB: Western blot of total proteins with the TaGID2 antibody; Co-IP: Western blot of the TaGID2 antibody for proteins captured by co-immunoprecipitating total proteins with the DELLA antibody.

Western blot and co-immunoprecipitation analyses demonstrated that the TaGID2 protein was approximately 26 kDa (Fig 6). The Western blot analysis of total proteins with the TaGID2 antibody showed that RHT-1s existed in all samples, suggesting that TaGID2s interact with RHT-1s in wheat. In addition, a Western blot analysis using the TaGID2 antibody for proteins captured by co-immunoprecipitating total proteins with the DELLA antibody also confirmed interaction between TaGID2s and RHT-1s in wheat. Moreover, common wheat is hexaploid with A, B, and D genomes in which most genes have three copies, and blots also existed in the mutant samples, such as RHT-B1b, RHT-B1e, RHT-B1k, and RHT-D1h, illustrating that the *Rht-1s-*encoded DELLA proteins were functionally redundant.

RHT-1s were localized in nuclei. Therefore, TaGID2s-GFP/YFP fusion genes were constructed to identify whether the interaction between TaGID2 and RHT-1 occurred in nuclei. Transient expression of TaGID2s-GFP and TaGID2s-YFP in onion epidermal cells (S4 Fig) and *Arabidopsis* mesophyll protoplasts (S4 Fig) revealed bright spots of GFP and YFP fluorescence only in nuclei, whereas that of the controls were found in nuclei and the cytoplasm. Thus, the TaGID2s were localized in the nuclei.

### Interaction between TaGID1-RHT1-TaGID2

Our previous study isolated three GA receptor genes called *TaGID1-A1*, *TaGID1-B1*, and *TaGID1-D1* from hexaploid wheat, and a yeast two-hybrid assay demonstrated that TaGID1s interact with the DELLA proteins RHT-A1a, RHT-B1a, and RHT-D1a in the presence of GA_3_[33]. Yeast two-hybrid and BiFC assays were carried out to detect the interactions between TaGID1 and 14 RHT-1 mutants *in vitro* and *in vivo* and identify the TaGID1 and RHT-1 interacting region. The yeast two-hybrid (S5 Fig) and BiFC (S6 Fig) assays showed that TaGID1 interacted with most RHT-1s in a GA-dependent manner, except RHT-B1b, RHT-B1e, RHT-B1k, and RHT-D1g. The interaction between TaGID1 and RHT-B1b/RHT-B1e failed because of a premature termination mutation in the DELLA domain, indicating that the interaction between TaGID1 and RHT-1 occurs in the DELLA domain. TaGID1 could not interact with RHT-B1k or RHT-D1g due to a frameshift mutation in the VHIID and PFYRE motifs leading to deficiency of the GRAS domain, demonstrating that the GRAS domain is required for DELLA protein function.

**Fig 7:**
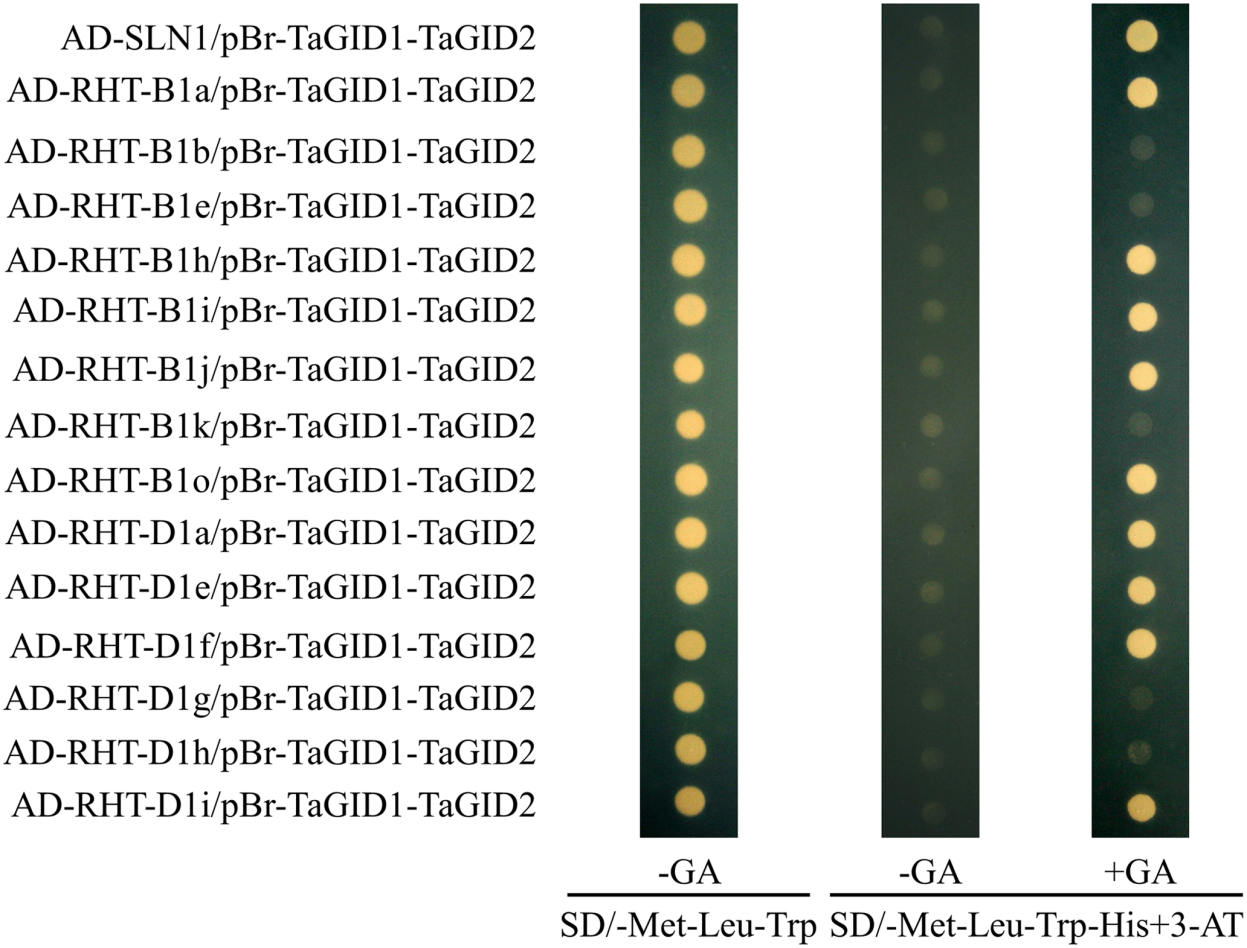
Interaction among TaGID1, TaGID2, and RHT-1 in yeast cells. SLN1 and RHT-1s were used as preys, pBr-TaGID1A-TaGID2A was used as baits, and the AD and pBr constructs were co-transformed into the yeast strain HF7c, the transformants were grown on the SD/-Met-Leu-Trp media (control) and the SD/-Met-Leu-Trp-His media containing 20 mM 3-AT with or without 100 μM GA_3_, all experiments were conducted three times, and five clones were used each time.

Because the yeast two-hybrid assay showed that TaGID1-A1 and TaGID2-A1 exhibited stronger affinity for DELLA proteins, the pBr-TaGID1A-TaGID2A expression vector was generated as the bait plasmid for the yeast three-hybrid assay with pGADT7-*RHT1*s as the prey plasmids. The pBr-TaGID1A-TaGID2A bait plasmid interacted with most RHT-1s, except RHT-B1b, RHT-B1e, RHT-B1k, RHT-D1g, and RHT-D1h in the presence of GA_3_ (Fig 7), demonstrating that TaGID1 was the GA receptor. TaGID1 and RHT-1 interacted with the RHT-1 protein DELLA domain after TaGID1 received the GA signal. The interaction induced TaGID2 to recognize the RHT-1 protein through the LHRII motif of the RHT-1 protein, and the GA-TaGID1-DELLA-TaGID2 complex was subsequently degraded via the ubiquitin-proteasome pathway, which was triggered from the interaction between TaGID2 and TSK1.

## Discussion

The specificity of ubiquitination, which is an essential post-translational modification that regulates signaling and protein turnover in eukaryotic cells, is driven by SCF ubiquitin E3 ligases. As a core component of the SCF complex, F-box proteins, named for their highly conserved F-box motif, recognize and bind to the substrate protein [13]. The F-box proteins SLY1 and SNE in *Arabidopsis* and GID2 in rice are involved in the GA signaling pathway [22, 24–25, 27–28, 40]. However, little information is available about homologous F-box proteins in wheat. Consequently, this study was carried out to genetically characterize and describe the functions of F-box proteins in wheat.

The F-box gene is conserved in many plant species, so identifying the *AtSLY1* and *OsGID2* genes greatly facilitated homologous cloning of *TaGID2* [22, 24, 27–28]. As common wheat is a hexaploid plant containing three sets of genomes, three genes, called *TaGID2-A1*, *TaGID2-B1*, and *TaGID2-D1*, were isolated from the Chinese Spring wheat variety by PCR-based cloning. A phylogenetic analysis of the GID2 proteins showed that TaGID2s had a close evolutionary relationship with OsGID2, suggesting that TaGID2s may play similar roles to those of OsGID2 in wheat. The deduced amino acid sequences of the three TaGID2s were highly consistent with OsGID2, particularly the conserved F-box domain, which functions as a protein-protein interaction site during formation of the E3 ubiquitin-ligase SCF complex [12–13]. Besides the N-terminal F-box motif, many F-box proteins contain another protein-protein interaction domain in their C-terminal, such as leucine-rich repeats (LRRs), WD-40 repeats, or Kelch. Hence, F-box proteins are grouped into three categories in humans and mice: FBXL denotes a protein containing an F-box and LRRs, FBXW denotes a protein with an F-box and WD repeats, and FBXO denotes a protein with an F-box and either another or no other motif [41–42]. Furthermore, Gagne et al. (2002) divided 694 potential *Arabidopsis* F-box proteins into five distinct families and 20 subfamilies by phylogenetic analysis [15]. However, we did not find any conserved motifs, such as FBXL, FBXW, or FBXO, outside the F-box domain in the structures of TaGID2s, but the structures of TaGID2s share the three conserved domains of the F-box, GGF, and LSL domains, with AtSLY1 and OsGID2. In addition, TaGID2 and OsGID2 both have a VR1 domain, which is absent in AtSLY1 [22, 28]. Gomi et al. (2004) demonstrated that the F-box, GGF, and LSL domains are essential for OsGID2 function, whereas the VR1 domain is not necessary, and the N-terminus region may not be important for OsGID2 function; thus, the domain differences between GID2 and SLY1 are negligible [28]. TaGID2s shared all domains with OsGID2, further supporting that they may play similar roles.

The three *TaGID2*s shared a similar expression pattern during the wheat heading stage, were expressed in all tested organs, and exhibited higher expression levels in young spikes and elongating stems, but lower expression levels in flag leaves and roots in accordance with *OsGID2*, which is highly expressed in stems, the shoot apex, and unopened flowers [28]. They were similar to the expression pattern of the *SD1* gene, which encodes a key enzyme in GA biosynthesis [43]. The expression analysis indicated that *TaGID2*s are involved in the GA response pathway. Moreover, GA downregulated the expression of *TaGID2*s, suggesting that *TaGID2*s are positive regulators of GA signaling, as described for OsGID2 and AtSLY1 [20–22, 24–28].

The F-box domain is essential for the interaction between OsGID2 and OsSkp15, resulting in formation of the SCF^GID2^ complex [28]. The *Arabidopsis ASK1* homologous gene *TSK1*, as described by Li et al. (2006), was cloned from the Chinese Spring wheat variety to confirm that TaGID2s actually encode the F-box protein and to clarify the role of TaGID2 in formation of the SCF complex. The *TSK1* gene we obtained had two SNPs compared with AY316293, which was cloned from *T*. *aestivum* cv. Jingdong 1. They may be the same gene in different cultivars or they could be different copies of the same gene because of the hexaploidy of wheat [31]. The yeast two-hybrid assay demonstrated that TaGID2s interacted directly with TSK1 in a GA-independent manner, as other F-box proteins; thus, TaGID2s formed an E3 ubiquitin-ligase SCF complex by interacting with TSK1 [15–17].

Transient expression of GFP and YFP confirmed that TaGID2s are localized in the nuclei, as described for AtSLY1 [24]. GA-independent interactions occurred between the three TaGID2s and RHT-A1a, RHT-B1a, and RHT-D1a, demonstrating that RHT-1s are TaGID2 targets. However, Gomi et al. (2004) revealed that SCF^GID2^ only targets the SLR1 protein under a phosphorylated state. This distinction needs further investigation of the GA signaling mechanism in wheat. The yeast two-hybrid assay, Western blot analysis, and co-immunoprecipitation results showed that TaGID2s interacted with RHT-1s. The yeast two-hybrid assay for TaGID2s and the 14 allelic variations of *Rht-1* showed that RHT-B1b and RHT-B1e cannot interact with TaGID2s due to their premature termination mutation in the DELLA motif. Dill et al. (2004) also demonstrated that SLY1 interacts directly with RGA and GAI via the C-terminus of the GRAS domain, suggesting that the GRAS domain is essential for RHT-1 protein-protein interactions. The frameshift mutation in RHT-B1k prevented the interaction with TaGID2s, but RHT-D1g, possessing another frameshift mutation, was able to interact with TaGID2s, and the missense mutation of RHT-D1h in the LHRII motif resulted in the loss of the DELLA-GID2 interaction, indicating that the TaGID2 and RHT-1 interaction occurs in the LHRII motif of the DELLA protein.

We demonstrated previously that the wheat TaGID1 GA receptor interacts with the RHT-A1a, RHT-B1a, and RHT-D1a DELLA proteins in the presence of GA_3_ [33]. In this study, we employed yeast two-hybrid, BiFC, and yeast three-hybrid assays to investigate the interactions between TaGID1-RHT1-TaGID2. The results showed that RHT-B1b and RHT-B1e did not interact with TaGID1 because of the stop codon, indicating that the TaGID1 and RHT-1 interaction occurred in the DELLA domain. However, as a result of the frameshift mutation in the VHIID and PFYRE motifs, TaGID1 did not interact with RHT-B1k or RHT-D1g, verifying that the GRAS domain is required for DELLA protein function.

RHT-1 interacted with the GA receptor TaGID1 in the DELLA domain of the RHT-1 protein after TaGID1 received the GA signal. This interaction induced TaGID2 to recognize the RHT-1 protein through the LHRII motif of the RHT-1 protein, and the E3 ubiquitin ligase SCF complex recognized the GA-TaGID1-RHT1-TaGID2 complex through the TaGID2 and TSK1 interaction, triggering degradation of RHT-1 via the ubiquitin-proteasome pathway. These findings are concordant with the well-known GA-GID1-DELLA model for the GA signaling pathway [6–11]. The identification and functional analysis of another GA receptor reported by Yano et al. (2015) and new proteins that interact with RHT-1s, will help us better understand the molecular mechanism of GA signal transduction in wheat [44].

## Supporting Information

S1 Fig Phylogenetic analysis of GID2 proteins in plants. OsGID2 (*Oryza sativa*, Q7XAK4), AtSLY1 (*Arabidopsis thaliana*, NP_194152), TaGID2L (*Triticum aestivum*, ABK79908), AetGID2 (*Aegilops tauschii*, EMT28630), BdGID2-like (*Brachypodium distachyon*, XP_003575230), SiGID2-like (*Setaria italica*, XP_004952896), ZmGID2 (*Zea mays*, NP_001149408), VvGID2-like (*Vitis vinifera*, XP_003632510), RcGID2 (*Ricinus communis*, XP_002510145), GmGID2-like (*Glycine max*, XP_003550317), BnSLY1 (*Brassica napus*, ACV88719), CsGID2-like (*Cucumis sativus*, XP_004163240), CaGID2-like (*Cicer arietinum*, XP_004501547), FvGID2-like (*Fragaria vesca*, XP_004291072), HaSLY1 (*Helianthus annuus*, ADO61003), LjSLY1a (*Lotus japonicus*, BAH78716), SlGID2-like (*Solanum lycopersicum*, XP_004238120), SmGID2a (*Selaginella moellendorffii*, ABX10760), SmGID2c (*S*. *moellendorffii*, ABX10761).
(DOC)

S2 Fig Nucleotide sequence alignment of the *TaGID2* genes. Three *TaGID2s* were amplified from cDNA and genomic DNA of wheat cultivar ‘Chinese Spring’. The red boxes show the location of the start (ATG) and stop (TGA) codons, respectively.
(DOC)

S3 Fig GID2s in common wheat and the wild diploid relatives. A) Amino acid sequence alignment of the GID2s from wheat and the wild diploid relatives, B) Phylogenetic tree of the GID2s from wheat and the wild diploid relatives.
(DOC)

S4 Fig The subcellular location of TaGID2s. A) Onion epidermal cells (Bar = 50 μm), B) *Arabidopsis* mesophyll protoplast cells (Bar = 10μm).
(DOC)

S5 Fig Interaction of TaGID1 and RHT-1 in yeast cells. TaGID1s were, from left to right, TaGID1-A1, TaGID1-B1, and TaGID1-D1 used as baits; SLN1 and RHT-1s were used as preys. The AD and BK constructs were co-transformed into the yeast strain AH109, then transformants grew on the SD/-Leu-Trp media (control) and the SD/-Leu-Trp-His-Ade media which supplemented with or without 100 μM GA_3_. All experiments were conducted three times, and five clones were used each time.
(DOC)

S6 Fig Interaction of TaGID1 and RHT-1 in *Arabidopsis* mesophyll protoplast cells (Bar = 10 μm). (DOC)
