# Supplementary material for: Molecular Characterization of Three GIBBERELLIN-INSENSITIVE DWARF2 Homologous Genes in Common Wheat
Source: PLoS One. 2016 Jun 21;11(6):e0157642. doi: 10.1371/journal.pone.0157642 (PMC4915692; doi:10.1371/journal.pone.0157642)
Supplement: S1 Fig — OsGID2 (Oryza sativa, Q7XAK4), AtSLY1 (Arabidopsis thaliana, NP_194152), TaGID2L (Triticum aestivum, ABK79908), AetGID2 (Aegilops tauschii, EMT28630), BdGID2-like (Brachypodium distachyon, XP_003575230), SiGID2-like (Setaria italica, XP_004952896), ZmGID2 (Zea mays, NP_001149408), VvGID2-like (Vitis vinifera, XP_003632510), RcGID2 (Ricinus communis, XP_002510145), GmGID2-like (Glycine max, XP_003550317), BnSLY1 (Brassica napus, ACV88719), CsGID2-like (Cucumis sativus, XP_004163240), CaGID2-like (Cicer arietinum, XP_004501547), FvGID2-like (Fragaria vesca, XP_004291072), HaSLY1 (Helianthus annuus, ADO61003), LjSLY1a (Lotus japonicus, BAH78716), SlGID2-like (Solanum lycopersicum, XP_004238120), SmGID2a (Selaginella moellendorffii, ABX10760), SmGID2c (S. moellendorffii, ABX10761). (DOC) [file pone.0157642.s001.doc]

**S1 Fig**


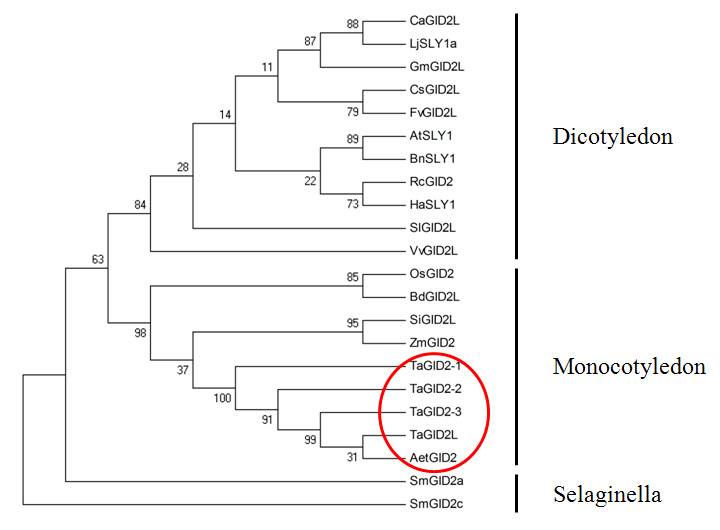


**S1 Fig. Phylogenetic analysis of GID2 proteins in plants.**

OsGID2 (*Oryza sativa*,Q7XAK4), AtSLY1 (*Arabidopsis thaliana*, NP_194152), TaGID2L (*Triticum aestivum*, ABK79908), AetGID2 (*Aegilops tauschii*, EMT28630), BdGID2-like (*Brachypodium distachyon*, XP_003575230), SiGID2-like (*Setaria italica*, XP_004952896), ZmGID2 (*Zea mays*, NP_001149408), VvGID2-like (*Vitis vinifera*, XP_003632510), RcGID2 (*Ricinus communis*, XP_002510145), GmGID2-like (*Glycine max*, XP_003550317), BnSLY1 (*Brassica napus*, ACV88719), CsGID2-like (*Cucumis sativus*, XP_004163240), CaGID2-like (*Cicer arietinum*,XP_004501547), FvGID2-like (*Fragaria vesca*, XP_004291072), HaSLY1 (*Helianthus annuus*, ADO61003), LjSLY1a (*Lotus japonicus*, BAH78716), SlGID2-like (*Solanum lycopersicum*, XP_004238120), SmGID2a (*Selaginella moellendorffii*, ABX10760), SmGID2c (*S. moellendorffii*, ABX10761).
