# Supplementary material for: Molecular Characterization of Three GIBBERELLIN-INSENSITIVE DWARF2 Homologous Genes in Common Wheat
Source: PLoS One. 2016 Jun 21;11(6):e0157642. doi: 10.1371/journal.pone.0157642 (PMC4915692; doi:10.1371/journal.pone.0157642)
Supplement: S2 Fig — Three TaGID2s were amplified from cDNA and genomic DNA of wheat cultivar ‘Chinese Spring’. The red boxes show the location of the start (ATG) and stop (TGA) codons, respectively. (DOC) [file pone.0157642.s002.doc]

**S2 Fig**


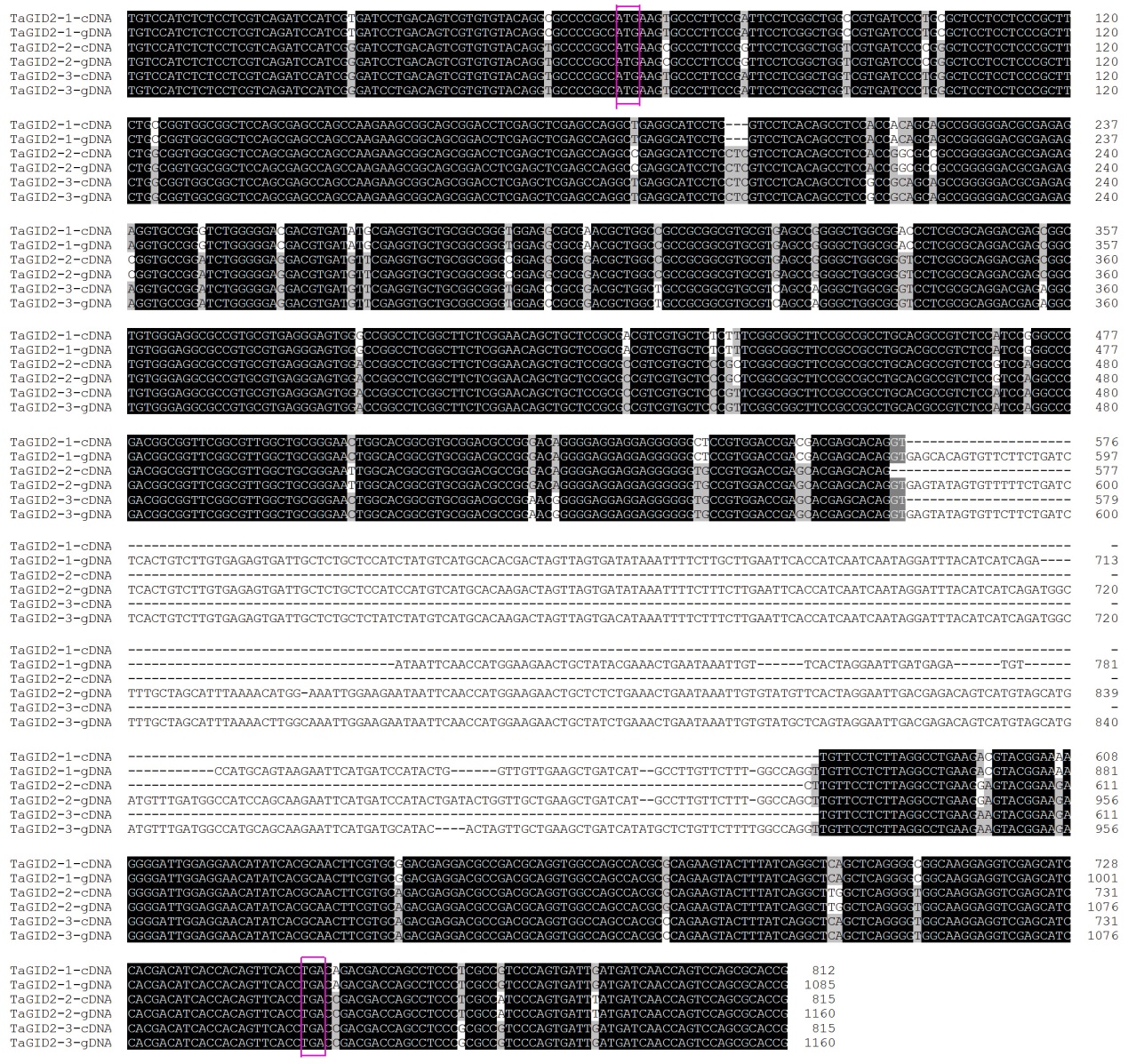


**S2Fig. Nucleotide sequence alignment of the *TaGID2* genes.**

Three *TaGID2s* were amplified from cDNA and genomic DNA of wheat cultivar ‘Chinese Spring’. The red boxes show the location of the start (ATG) and stop (TGA) codons, respectively.
