# Supplementary material for: Molecular Characterization of Three GIBBERELLIN-INSENSITIVE DWARF2 Homologous Genes in Common Wheat
Source: PLoS One. 2016 Jun 21;11(6):e0157642. doi: 10.1371/journal.pone.0157642 (PMC4915692; doi:10.1371/journal.pone.0157642)
Supplement: S3 Fig — A) Amino acid sequence alignment of the GID2s from wheat and the wild diploid relatives, B) Phylogenetic tree of the GID2s from wheat and the wild diploid relatives. (DOC) [file pone.0157642.s003.doc]

**S3 Fig**

**
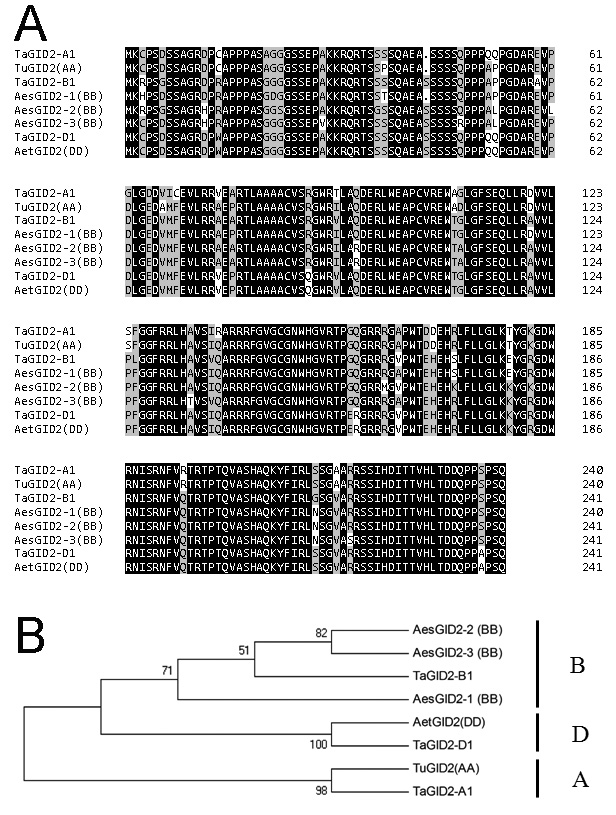
**

**S3 Fig. GID2s in common wheat and the wild diploid relatives.**

(A) Amino acid sequence alignment of the GID2s from wheat and the wild diploid relatives, (B) Phylogenetic tree of the GID2s from wheat and the wild diploid relatives.
