# Supplementary material for: Molecular Characterization of Three GIBBERELLIN-INSENSITIVE DWARF2 Homologous Genes in Common Wheat
Source: PLoS One. 2016 Jun 21;11(6):e0157642. doi: 10.1371/journal.pone.0157642 (PMC4915692; doi:10.1371/journal.pone.0157642)
Supplement: S4 Fig — A) Onion epidermal cells (Bar = 50 μm), B) Arabidopsis mesophyll protoplast cells (Bar = 10μm). (DOC) [file pone.0157642.s004.doc]

**S4 Fig**


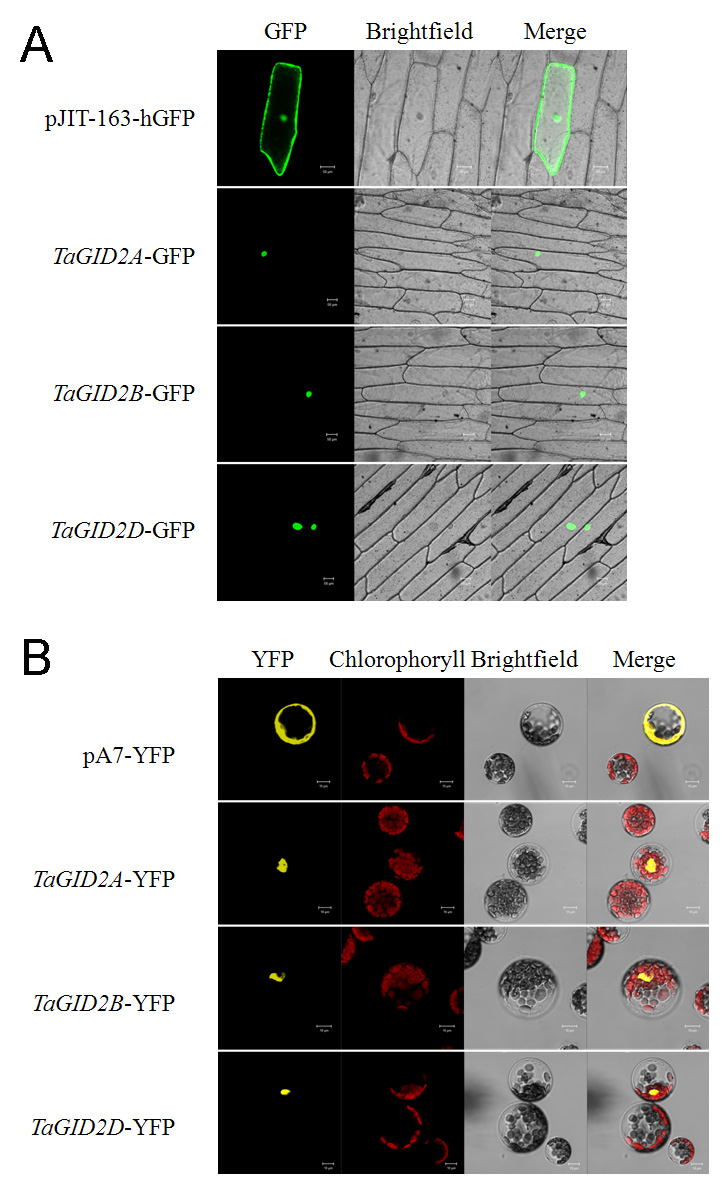


**S4 Fig. The subcellular location of TaGID2s.**

(A) Onion epidermal cells (Bar = 50 μm), (B) *Arabidopsis* mesophyll protoplast cells (Bar = 10μm).
