# Supplementary material for: Molecular Characterization of Three GIBBERELLIN-INSENSITIVE DWARF2 Homologous Genes in Common Wheat
Source: PLoS One. 2016 Jun 21;11(6):e0157642. doi: 10.1371/journal.pone.0157642 (PMC4915692; doi:10.1371/journal.pone.0157642)
Supplement: S5 Fig — TaGID1s were, from left to right, TaGID1-A1, TaGID1-B1, and TaGID1-D1 used as baits; SLN1 and RHT-1s were used as preys. The AD and BK constructs were co-transformed into the yeast strain AH109, then transformants grew on the SD/-Leu-Trp media (control) and the SD/-Leu-Trp-His-Ade media which supplemented with or without 100 μM GA3. All experiments were conducted three times, and five clones were used each time. (DOC) [file pone.0157642.s005.doc]

**S5 Fig**

**
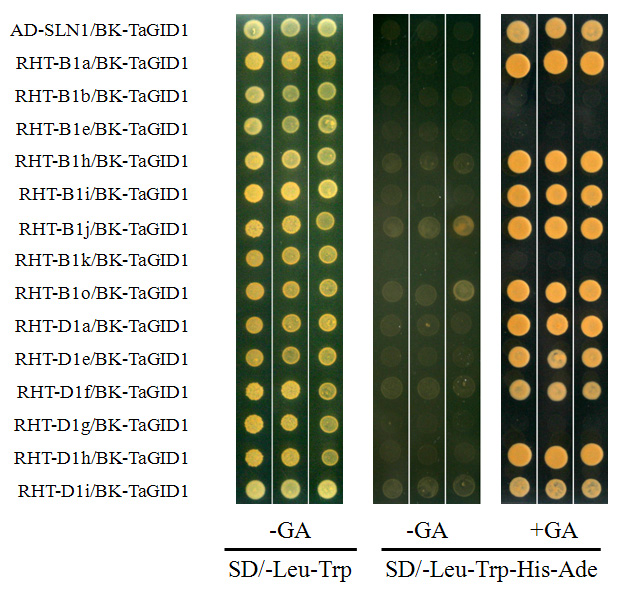
**

**S5 Fig. Interaction of TaGID1 and RHT-1 in yeast cells.**

TaGID1s were, from left to right, TaGID1-A1, TaGID1-B1, and TaGID1-D1 used as baits; SLN1 and RHT-1s were used as preys. The AD and BK constructs were co-transformed into the yeast strain AH109, then transformants grew on the SD/-Leu-Trp media (control) and the SD/-Leu-Trp-His-Ade media which supplemented with or without 100 μM GA_3_. All experiments were conducted three times, and five clones were used each time.
