# Supplementary figures and images for: Molecular Characterization of Three GIBBERELLIN-INSENSITIVE DWARF2 Homologous Genes in Common Wheat
Source: PLoS One. 2016 Jun 21;11(6):e0157642. doi: 10.1371/journal.pone.0157642 (PMC4915692; doi:10.1371/journal.pone.0157642)

**S6 Fig**


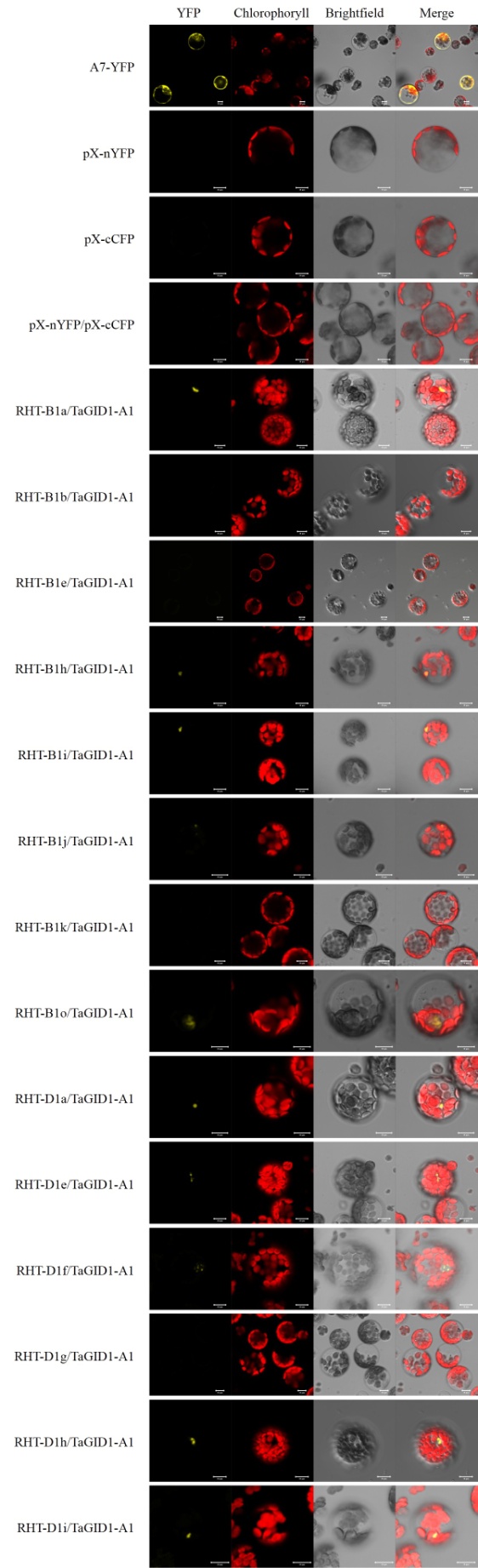


**S6 Fig. Interaction of TaGID1 and RHT-1 in*Arabidopsis* mesophyll protoplast cells (Bar = 10 μm).**

Supplement: S6 Fig — (DOC) [file pone.0157642.s006.doc]
